# Non-pharmacological labor pain management and associated factor among skilled birth attendants in Amhara Regional State health institutions, Northwest Ethiopia

**DOI:** 10.1186/s12978-020-01043-1

**Published:** 2020-11-23

**Authors:** Almaz Aklilu Getu, Simegnew Asmer Getie, Getahun Belay Gela, Eleni Admassu Maseresha, Birhanu Elifu Feleke, Alemtsehay Mekonen Muna

**Affiliations:** 1 Department of Midwifery, College of Medicine and Health Science, BahirDar University, BahirDar, Ethiopia; 2 School of Public Health, College of Medicine and Health Science, BahirDar University, BahirDar, Ethiopia

**Keywords:** Non-pharmacological, Pain management, Skilled birth attendant, Ethiopia

## Abstract

**Background:**

Healthcare providers have a responsibility to provide pain management support to women during labor. Labor pain management in low and middle income countries primarily relies on non-pharmacological methods, as there is little access to pharmacologic pain management. This study aimed to determine the utilization of non-pharmacological labor pain management (NPLPM) and associated factors among skilled birth attendants (SBAs) in Amhara Regional State health institutions, Ethiopia.

**Methods:**

A cross-sectional study was conducted on 592 SBAs working in the Amhara Region, Ethiopia. A multistage sampling was used to collect data using a pretested interview-administered questionnaire. Descriptive analysis was done to characterize the study population. Logistic regression was used to model predictors of NPLPM utilization among SBAs, including age, qualifications, type of medical institution, knowledge, attitudes, and the presence of a protocol.

**Result:**

Nearly forty seven percent 277(46.8%) of SBAs in the study cohort utilized NPLPM. SBAs who had adequate knowledge of NPLPM had 2.8 times increased odds of using NPLPM than SBAs who had inadequate knowledge. (95%CI 1.89–4.014). SBAs who had a positive attitude had 4.12 times increased odds of using NPLPM than SBAs with a negative attitude (95%CI 2.36, 7.2). SBAs who had labor a pain management protocol in their facility had 3.98 times increased odds of using NPLPM than those who didn’t have a labor pain management protocol (95%CI 1.83, 8.62).

**Conclusions:**

The analysis pointed to a gap in the utilization of NPLPM in the Amhara Region facilities studied. Less than half of SBAs used NPLPM when caring for laboring women. Professional factors related to use of NPLPM included the age of SBAs, their attitudes, level of education, and knowledge concerning pain management. NPLPM was also significantly associated with the availability of labor pain management protocols.

## Background

Labour pain is a universal concern for women. Addressing pain relief during childbirth is a way of promoting a satisfactory birth experience and a healthy reproductive outcome for women [1, 2, 4]. Non-pharmacological labor pain management (NPLPM) methods are noninvasive, cheap, simple [4–6], effective for prevention of postpartum depression [7–9] and postpartum hemorrhage [10] along with increasing maternal satisfaction [11, 12].

In many developed countries, pain relief in labor is considered as a routine part of intrapartum care and all women have access to the method of pain relief that they choose. However, in developing countries, including Ethiopia, options for labor pain alleviation are very limited [12–17] particularly in the Amhara region where there is no utilization of pharmacologic labor pain management techniques [18].

In previous study the possible barrier was not properly identified additionally it was done only on referral hospitals. This study, expands upon the published research documenting poor utilization of labor pain management across regional referral facilities [18]. This research was conducted to determine the utilization of non-pharmacological labor pain management and associated factors in all type of governmental health institutions in the Amhara Region, Ethiopia.

## Methods

An institution based cross-sectional study design was implemented among skilled birth attendants who were working in Amhara Region governmental health institutions from May 1 to 30 2019. Amhara Regional State is one of the nine regional states in Ethiopia. This region has a population of 21,134,988 of whom about 4,897,566 are women in reproductive age and the region have 51 hospitals and 839 health centers; among these five are referrals hospitals, four general and 42 primary hospitals. The hospitals have a total of 1023 skilled birth attendants. These hospitals have 646 beds for labor and delivery service and approximately 84,440 deliveries per year [19].The health centers have a total of 3023 skilled birth attendants and 12,900 deliveries per year. In this study, all health professionals who had been working in labor and delivery ward were included in the study. Those classified as skilled birth attendants included Obstetrician/Gynecologists, Residents, General Practitioners (GP’s), midwives, nurses, health officers and emergency surgeons.

The sample size was calculated using a single population proportion formula, by considering the following assumptions. The proportion (p) consider as 50%, 95% confidence level of Z = 1.96, 5% of absolute precision. By adding 5% for non-response and considering design effect 2 the final sample size was calculated to be 605.

Multistage sampling technique was applied. A stratified sampling technique was used to classify the health institutions into four categories (referral hospitals, general hospital, primary hospitals and health centers) which were considered on distinction of assigned professionals, service delivery and client flow as gynecologist/obstetrician was not assigned at primary hospitals and health centers in the region.

Among the four strata simple random sampling technique was employed to select out the eligible facilities: 3 referral hospitals out of 5, 2 general hospitals out of 4, and 20 primary hospitals out of 42 in the region. Fifty two health centers were randomly selected from the 839 eligible health centers. Finally, all skilled birth attendants in randomly selected hospitals and health centers were included in the study.

The study participants were all health professionals (gynecologist, residents, GP (general practitioner), midwives, nurses, health officers and emergency surgeons) working in labor and delivery ward. We excluded students (interns, midwifes and nurses) from the study.

Utilization of nonpharmacological labor pain management was the outcome variable whereas socio demographic characteristics, Health professional factors (knowledge, attitude) and Institutional factors (type of health institution, and availability of protocols) were taken to be independent variables.

Questionnaire was developed after an extensive review of the literature. The tool was modified and finalized according to the suggestions and recommendations of local experts. A pre-tested structured interview questionnaire was used for data collection. Five supervisors and ten Bachelor of Science (BSc) midwives were employed as data collectors. Training was given to data collectors and to the research supervisors. A pre-test was conducted with 30 skilled birth attendants randomly chosen from a population outside of the study area. Questionnaires were cleaned daily by data collection supervisors under the primary investigator’s oversight. Questionnaires were checked for completeness, consistency and when missing items were discovered, the items were collected and coded appropriately.

The collected data was checked for completeness and consistency by the supervisors under the guidance of the primary investigator. Data was cleaned, coded and entered into EPI data 3.1 and exported for analysis to SPSS version 20. Descriptive statistics were computed to describe data. Bivariable analysis was done and all variable with p < 0.2 was analyzed in multivariable logistic regression model to identify the association between explanatory and outcome variables. Adjusted Odds ratio **(**OR) with 95% CI was calculated in order to measure the strength of association between explanatory variables and the outcome variable, with the level of statistical significance set at p < 0.05.

## Result

### Socio-demographic characteristics

A total of five hundred ninety-two skilled birth attendants were enrolled in this study with a response rate of 97.8%. The mean age of respondents was 27.6 years with SD of 3.3 years of this majority. Over eight one percent (81.1%) of the participants were in the age range between 20 and 29 years. The majority of respondents were orthodox Christians (95.1%). More than half (60.1%) of the participants were male and majority (85.5%) of the participants were midwives followed by medical doctors.

Regarding the educational status more than half (54.1%) of respondents had BSc degrees and 10.2% of the skilled birth attendants had postgraduate degrees. The breakdown of facilities reported that respectively participants were from referral hospitals, general hospitals and primary hospitals (33.1%, 7.9% and 32.6% (Table 1).

**Table 1:** Distribution of professionals by their socio-demographic characteristic in ARS health institutions, Northwest Ethiopia, May 1–30, 2019 (n = 592)

Characteristics	Frequency (n)	Percent (%)
*Age*		
20–29	489	81.1
30–39	90	15.2
> 40	18	3
*Sex*		
Male	356	60.1
Female	236	39.9
*Religion*		
Orthodox	563	95.1
Muslim	26	4.4
Others*	3	.5
*Profession*		
Midwife	506	85.5
Medical Doctor	36	6.2
Nurse	26	4.4
Emergency surgery	24	4
*Level of education*		
Degree	320	54.1
Diploma	212	35.8
Master	29	4.9
Resident	24	4.1
Gynecologist	7	1.2
*Year of experience*		
1–5	361	61
6–9	208	35.1
> = 10	23	3.9
*Type of health institution*		
Referral hospital	196	33.1
General hospital	47	7.9
Primary hospital	193	32.6
Health center	156	26.4

^*^Protestant and catholic

### Knowledge and attitude of skilled birth attendants on non-pharmacological labor pain management

**Fig. 1:**
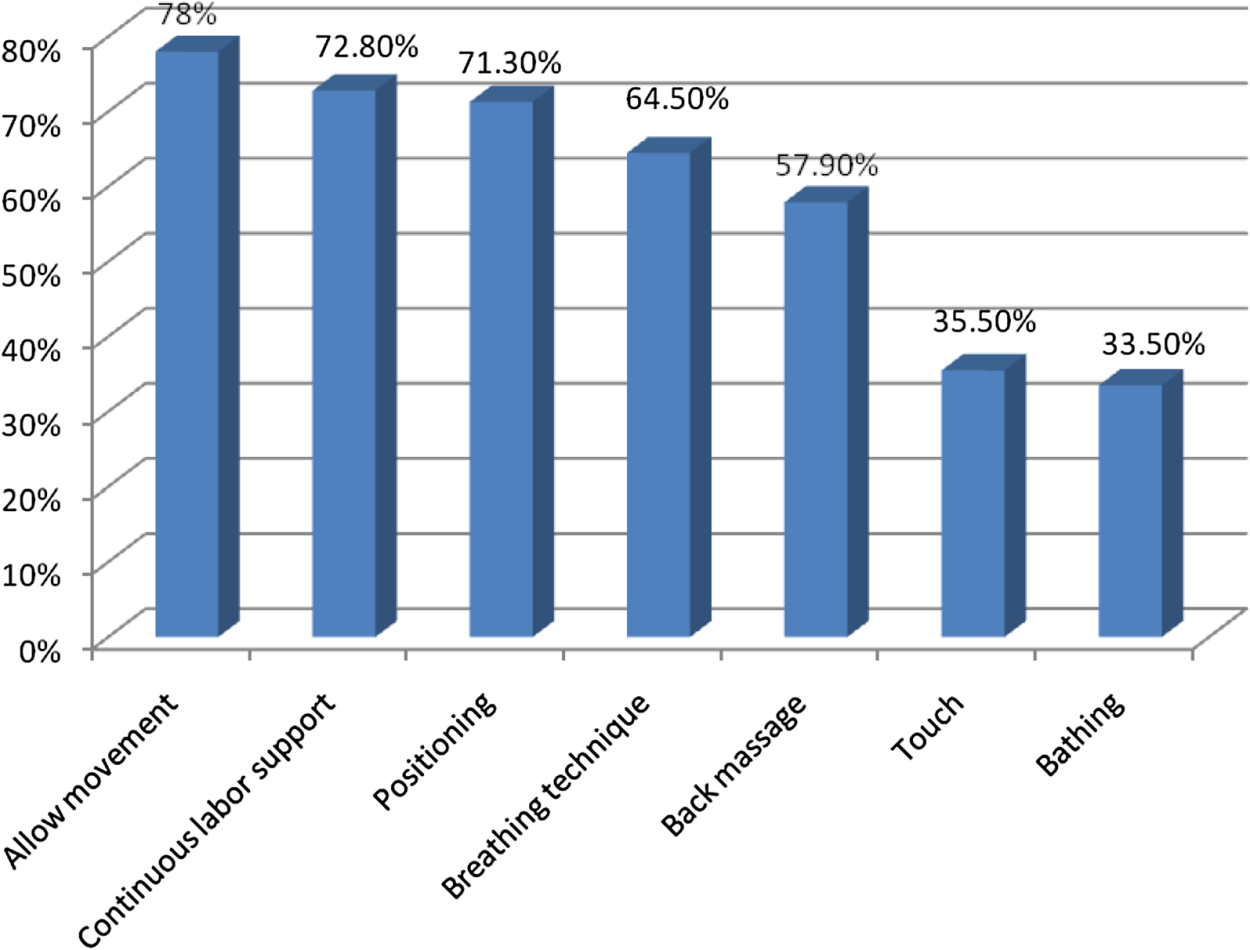
Professionals knowledge on types of NPLPM in ARSHI, Northwest Ethiopia, May 1–30, 2019 (n = 592)

**Fig. 2:**
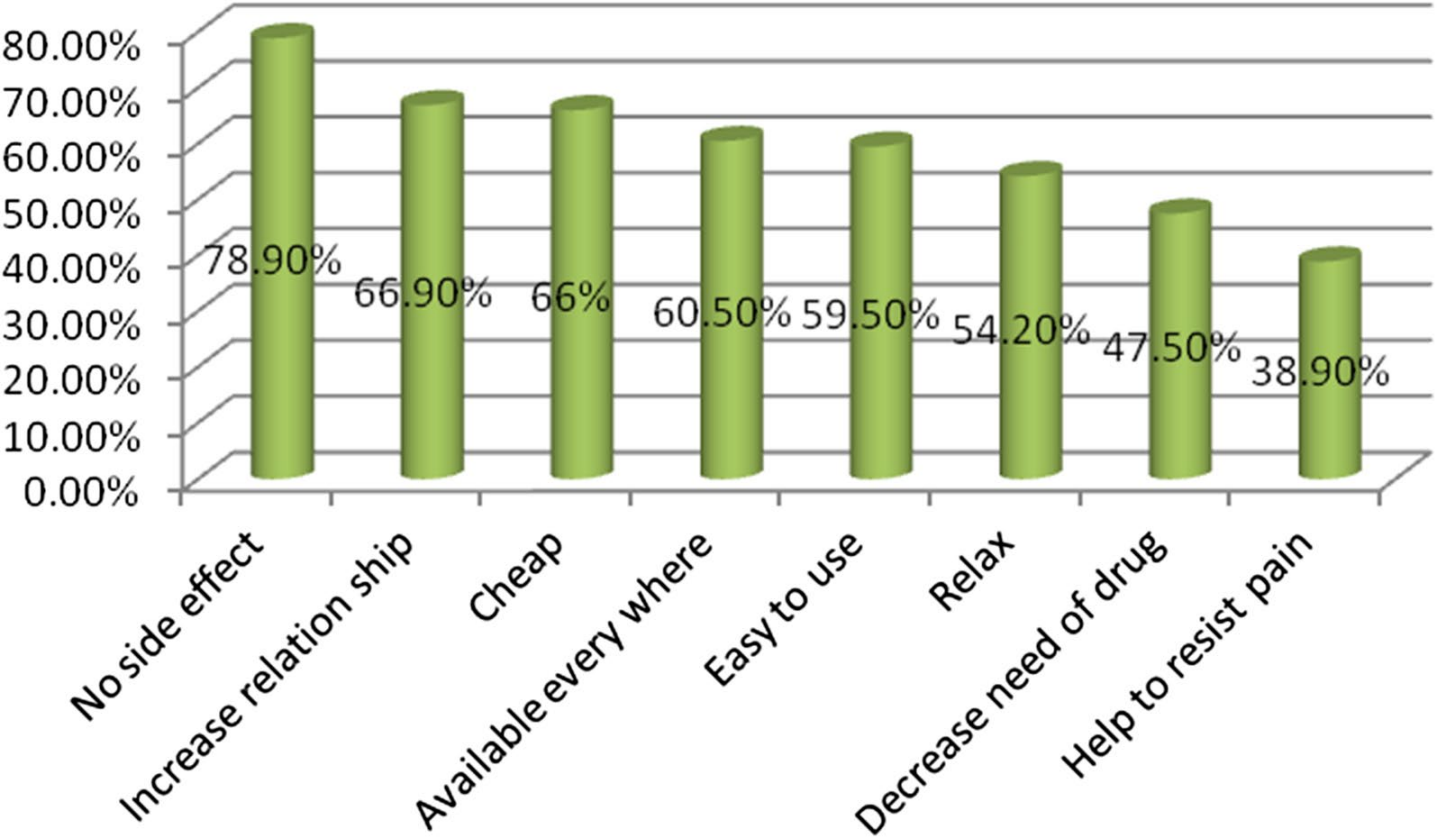
Professionals knowledge on the benefit of NPLPM in ARSHI, Northwest Ethiopia, May 1–30, 2019 (n = 592)

This study revealed that 541(91.4%) of respondents knew about NPLPM, with the most commonly known NPLPM methods being continuous labor support, allowing freedom of movement and positioning according to maternal preference (Fig. 1). Nonpharmacological labor pain management was also almost universally acknowledged by the skilled birth attendants as absent of adverse side effects (Fig. 2).

### Training in non-pharmacological pain management during labor

Approximately ninety six percent of the skilled birth attendants (95.5%) reported that they did not have any additional training in non-pharmacological labor pain management techniques. Less than one percent (0.5%) of the participants responded that they had received NPLPM training during their careers.

Regarding professional attitude on NPLPM, more than half of the 343 SBA’s (57.9%) believed that the women should resist labor pain which was explained by women was expected to cope labor pain by her selves considered as it was natural but 464(88.4%) of the providers recommended the use of NPLPM for laboring women in the same way 476(80.4%) of respondents believed that pain relief in labor was necessary.

### Utilization of non-pharmacological labor pain management

**Fig. 3:**
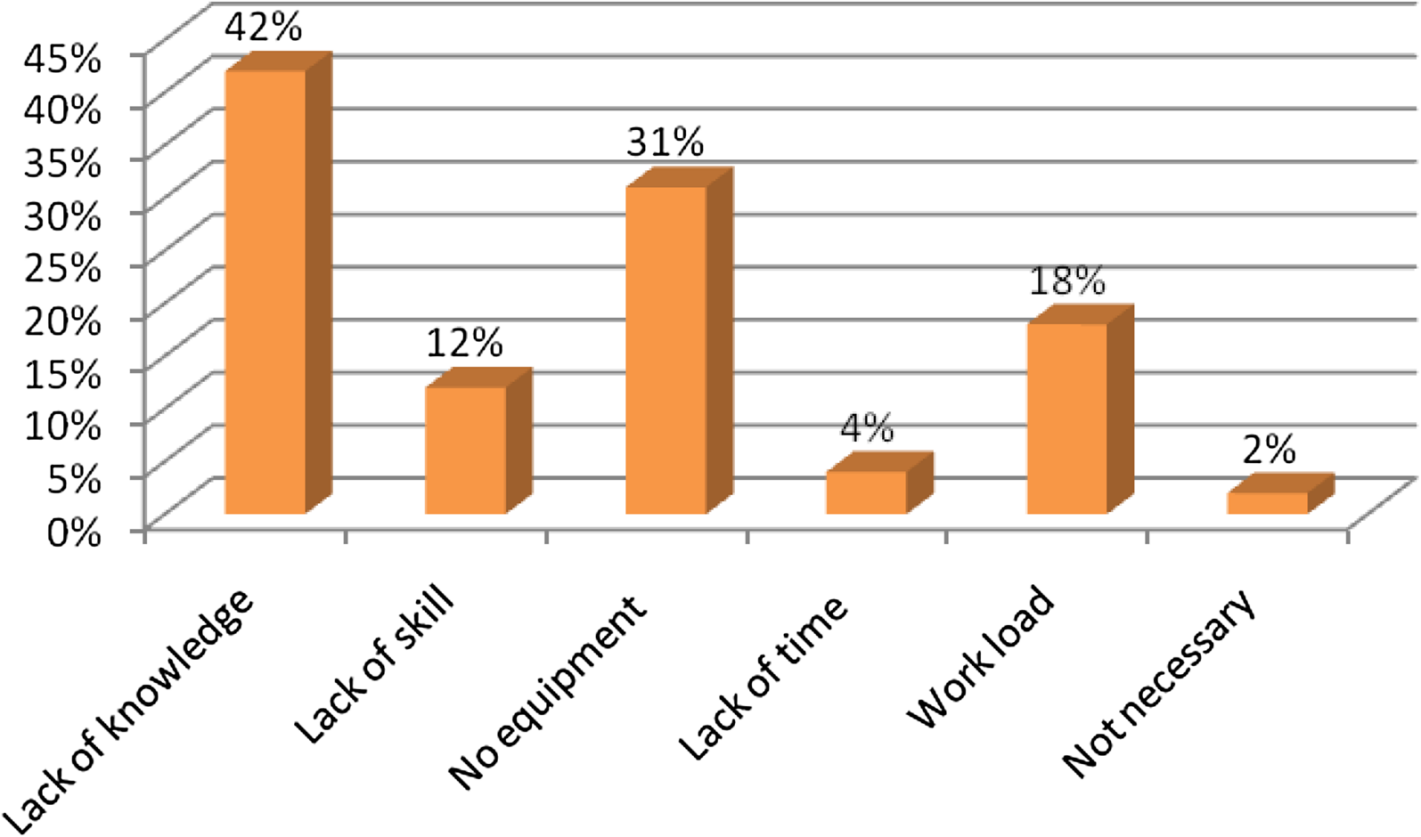
Reason of skilled birth attendants for non utilization of non-pharmacological labor pain management in ARSHI, Northwest Ethiopia, May 1–30, 2019 (n = 315)

Among 592 respondents 420(70.9%) thought that NPLPM was the best method of labor pain management. Almost thirty percent of the providers (n = 172; 29.1%) favored pharmacologic pain management. In this study, utilization of non-pharmacological labor pain management was utilitized by 46.8% of providers in Amhara Region’s State hospital institutions. The most commonly used NPLPM method was continuous labor support (76.6%) followed by allowing movement (ambulation) and relaxation 70.3%, 60.3% respectively. Lack of knowledge (42%) was among the most common reason for providers not utilizing NPLPM (Fig. 3).

### Factors associated with utilization of NPLPM

In the bivariate analysis, professional age, type of health institution, professional qualification, level of knowledge, attitude and presence of labor pain management protocol were significant at p-value < 0.05 level of significance. Among these, professional age, level of knowledge, attitude and presence of labor pain management protocol remained significant in the multivariable model.

Professional age 20–29 had 4.9 times greater odds of utilization of NPLPM compared to professional age 30 and older. {AOR = 4.9, 95% CI = (2.24−10.77)},those professionals who had adequate knowledge on NPLPM had 2.8 times increased odds of utilizing NPLPM than those with inadequate knowledge {AOR = 2.8, 95% CI = (1.89–4.14)}, similarly professionals who had positive attitude on NPLMPM had 4.1 times increased odds of utilizing NPLPM compared to professionals with negative attitude {AOR = 4.12, 95% CI = (2.36–7.2)} and those with a labor pain management protocol in their health institution had 3.9 times increased odds of utilizing NPLPM than their counterparts. {AOR = 3.98, 95% CI = (1.83–8.62)} (see Table 2).

**Table 2:** Bivariate and multivariate analysis of factors associated with utilization of NPLPM in ARSHI, Northwest Ethiopia, May 1–30, 2019 (n = 592)

	Utilization of NPLPM		COR (95%CI)	AOR (95%CI)
	Yes (%)	No (%)		
*Age*				
20–29	268 (96.7%)	252 (80%)	7.4 (3.62, 15.28)	4.92 (2.24, 10.77)**
> = 30	9 (3.2%)	63 (20%)	1	1
*Qualification®*				
Lower	90 (35.2)	122 (38.7%)	1	1
medium	164 (64.3)	156 (49.5%)	1.42 (1.01,2.02)	1.59 (1.06,2.41)
Higher	23 (9%)	37 (11.7%)	0.84 (0.47,1.52)	0.66 (0.33,1.31)**
*Type of institution*				
Referral hospital	85 (30.6)	111 (35.2%)	0.81 (0.53,1.23)	0.63 (0.37,1.04)
General hospital	15 (5.4%)	32 (10.1%)	0.49 (0.25,0.98)	0.47 (0.22,1.01)
Primary hospital	101 (36.4%)	92 (29.2%)	1.16 (0.76,1.76)	1.17 (0.72,1.93)
Health center	76 (27.4%)	80 (25.3%)	1	1
*Knowledge*				
Adequate	171 (61.7%)	101 (32%)	3.41 (2.43,4.79)	2.8 (1.89,4.14)**
Inadequate	106 (38.2%)	214 (67.9%)	1	1
*Attitude*				
Positive	252 (90.9%)	207 (65.7%)	5.26 (3.28,8.43)	4.12 (2.36,7.2)**
Negative	25 (9%)	108 (34.2%)	1	1
*Availability of protocol*				
Yes	29 (10.4%)	13 (4.1%)	2.71 (1.38,5.34)	3.98 (1.83,8.62)**
No	248 (89.5%)	302 (95.8%)	1	1

**®**Lower level: diploma in midwifery, medium level: BSc in midwifery and general practitioner, higher level: MSc, obstetrician and resident

## Discussion

This study tried to determine utilization of non-pharmacological labor pain management which was carried out for the first time in all level of governmental health institutions and identify barriers for its use in ARSHI, North West Ethiopia.

The present study established that the proportion of skilled attendants utilizing non-pharmacologic labor pain management methods were 46.8%. This study finding is in line with the study done in Tigray (Ethiopia) and Egypt that was 43.3% and 44.9% respectively [20, 21]. However, it is higher than the study done in Ethiopia which was 40.1% [18].This may be due to a difference in the study period, level of health institution and sample size, this study was conducted only in referral hospitals but the current study includes all levels of the health institution.

The current study found that one of the barriers to poor utilization of NPLPM was inadequate knowledge of skilled birth attendants about NPLPM. SBA who have adequate knowledge of NPLPM was 2.8 times more likely to use the NPLPM method than SBA who have inadequate knowledge. {AOR (95%CI) = 2.8(1.89–4.014)} similarly lack of knowledge is one of the barriers for the use of NPLPM in Kenya and Nigeria [22, 23].

Professional who have a positive attitude is 4.12 times more likely to give NPLPM than SBA with negative attitude.{AOR(95%CI) = 4.12 (2.36,7.2)} in this study 80.4% of respondents believed that pain relief in labor is necessary which is similar to study done in Egypt and India 78% and 92% respectively [21, 24].

Based on a current study there is a significant difference in the level of qualification for the utilization of NPLPM being a high-level profession is a protection for the utilization of NPLPM. This difference may not be due to the variation in their level of knowledge on NPLPM instead this may due to the most highly qualified SBAs not follow laboring mother instead they may call for a consultation. This finding is supported by different studies done in Ethiopia, Bangladesh and Australia [18, 25, 26]. The current study revealed that 72% of midwives prefer the use of NPLPM rather only 55% of obstetrician prefer the use of NPLPM similarly obstetrician in Australia had a personal preference on pharmacological pain relief methods.

In this study availability of labor pain management protocol was a statistically significant predictor of utilization of NPLPM which was primarily identified by this study. SBA who had labor pain management protocols in their facility had 3.98 times more likely to use NPLPM than SBA who didn’t have labor pain management protocol. {AOR (95%CI) = 3.98 (1.83, 8.62)} similarly unavailability of labor pain protocol was one of the barrier for the use of labor pain management in Tanzania [27].

The findings of this study should be viewed in light of the following limitations. The study population included only skilled birth attendant’s who provide routine maternity care it excluded cadres like hospital, zonal and regional health managers who may have different experience and attitude towards the practice of labor pain management which is necessary when addressing barriers to implement good quality of care including pain relief additionally the study did not investigate the women’s views about pain relief in labor.

## Conclusion

The management of the mother’s pain in labor is uncomprehensive. Professional age, knowledge, attitude, level of education and availability of labor pain management protocol were found significantly associated with the practice of non-pharmacologic labor pain management. The main barrier for poor utilization of NPLPM was the lack of knowledge, negative attitude and unavailability of pain management protocol.

## Recommendation

Efforts need to be done to increase the awareness and attitude of SBAs about NPLPM through short term training.

Availability and use of labor pain management protocol to be considered as essential component of maternity care.

## Data Availability

The data sets generated during the study are available from the corresponding author upon request.

## Abbreviations

ARSHIs: Amhara Region State Health Institutions
FMOH: Federal Ministry of Health
GP: General practitioner
HCPs: Health care providers
IDI: In-depth interview
NPLPM: Non pharmacological labor pain management
RHs: Referral hospitals
SBA: Skilled birth attendant
SWI: Sterile water injection
TENS: Transcutaneous electrical nerve stimulation
WHO: World Health Organization
